# In Vivo Tissue Distribution and Pharmacokinetics of FITC-Labelled *Hizikia fusiforme* Polyphenol–Polysaccharide Complex in Mice

**DOI:** 10.3390/foods13183019

**Published:** 2024-09-23

**Authors:** Yutong Li, Shangkun Li, Di Li, Yuan Gao, Shuhua Kong, Jingyi Liu, Shu Liu, Yichao Ma, Hui Zhou, Dandan Ren, Qiukuan Wang, Yunhai He

**Affiliations:** 1 College of Food Science and Engineering, Dalian Ocean University, Dalian 116023, China; 13848442033@163.com (Y.L.); 18736192663@163.com (S.L.); ldiss42@163.com (D.L.); 17853545180@163.com (Y.G.); kkkongshuhua@163.com (S.K.); 18623901902@163.com (J.L.); liushu@dlou.edu.cn (S.L.); 18640876812@163.com (Y.M.); zhouhui@dlou.edu.cn (H.Z.); rdd@dlou.edu.cn (D.R.); wqk320@dlou.edu.cn (Q.W.); 2 Key Laboratory of Aquatic Product Processing and Utilization of Liaoning Province, Dalian Ocean University, Dalian 116023, China; 3 National R&D Branch Center for Seaweed Processing, Dalian Ocean University, Dalian 116023, China

**Keywords:** *Hizikia fusiforme*, polyphenol–polysaccharide complex, fluorescent labelling, pharmacokinetics, tissue distribution

## Abstract

In this study, a quantitative method based on fluorescein isothiocyanate (FITC)-labelled *Hizikia fusiforme* polyphenol–polysaccharide complex (HPC) and its purified fractions (PC1, PC4) was used, and its pharmacokinetics and tissue distribution were investigated in mice. The results showed that the FITC-labelled method had good linearity (R^2^ > 0.99), intra-day and inter-day precision (RSD, %) consistently lower than 15%, recovery (93.19–106.54%), and stability (RSD < 15%), which met the basic criteria for pharmacokinetic studies. The pharmacokinetic and tissue distribution results in mice after administration showed that all three sample groups could enter the blood circulation. and HPC-FITC had a longer half-life (T_1/2_: 26.92 ± 0.76 h) and mean retention time (MRT_0–∞_: 36.48 h) due to its larger molecular weight. The three groups of samples could be absorbed by the organism in a short time (0.5 h) mainly in the stomach and intestine; the samples could be detected in the urine after 2 h of administration indicating strong renal uptake, and faecal excretion reached its maximum at 12 h. The samples were also detected in the urine after 2 h of administration. This study provides some theoretical basis for the tissue distribution pattern of polyphenol–polysaccharide complex.

## 1. Introduction

*Hizikia fusiformis*, an edible brown algae that grows on intertidal rocks along the northwestern Pacific coastline, belongs to the class *Sargassum*, order *Sargassum*, and family *Sargassoideae* [1]. In recent years, several studies have isolated and identified a variety of compounds with diverse pharmacological properties from *Hizikia fusiformis*, including polysaccharides [2,3], phenolic compounds [4,5], fucoxanthin, and fucoidan [6,7,8]. These compounds exhibit a range of effects, such as antioxidant [9], anti-inflammatory [10], hypoglycemic [11], hypolipidemic [12], and anti-aging [13] activities. However, the nutritional and commercial potential of *Hizikia fusiformis* remains underexploited.

Among the various bioactive compounds in algae, phenols have garnered significant attention due to their superior free radical scavenging activity. However, phenolic compounds are structurally unstable, whereas algal polysaccharides possess a stable structure but exhibit lower activity than polyphenols [14]. With advancing research on polyphenols and polysaccharides, natural polyphenol–polysaccharide complexes have been extracted from terrestrial plants. Magdalena et al. obtained polyphenol–polysaccharide complex from the flowers of *Sanguisorba officinalis* L., *Erigeron canadensis* L., and from *Fragaria vesca* L. and *Rubus plicatus*, which were able to protect human peripheral blood mononuclear cells (PBMCs) from γ-irradiation damage while maintaining the radiosensitivity of the myelogenous leukemia K562 cell line [15]. Joy et al. demonstrated that polyphenol–polysaccharide complex extracted from sprouted quinoa yogurt was effective in promoting the release of GLP-1 from NCI-H716 cells [16]. Currently, studies on polyphenol–polysaccharide complexes have predominantly focused on terrestrial medicinal plants, with research mainly concentrating on their isolation, purification, structural identification, and biological activity. However, there is a lack of research on their metabolic absorption patterns in vivo [17].

Recent studies have shown that fluorescent labelling [18], radioisotope labelling [19], and other methods can improve the sensitivity and specificity of in vivo detection of polysaccharides. Fluorescein isothiocyanate (FITC) is a fluorescence-based detection technique that has been widely used for drug microanalysis because of its specificity, sensitivity, and low-detection threshold [20]. Xu et al. studied FITC-labelled fucoidan and examined its ability to pass through a monolayer of Caco-2 cells, and the results showed that the FITC fucodian was detected at concentrations up to 1000 μg/mL and did not affect cell proliferation, indicating no toxic effect [21]. Fei et al. investigated the cytotoxicity of Cy5.5-labelled *Cucurbita moschata* polysaccharide (PPc-Cy5.5) on Caco-2 and RIN-m5F cells for 36 h as measured by CCK-8 assay. The results were evaluated and showed that cell survival was greater than 95% at different concentrations of PPc-Cy5.5. indicating that the preparation is safe [22]. The fluorescent labelling method has less effect on the biological activity of polysaccharides and is not cytotoxic [23]. Pharmacokinetics can quantitatively study the pattern of change during drug metabolism such as drug absorption, tissue distribution, metabolism, and elimination [24]. Currently, most pharmacokinetic studies have focused on polysaccharides [25] or polyphenols [26], but there is no standardised method for quantitatively detecting the pharmacokinetics and tissue distribution of polyphenol–polysaccharide complex in vivo. This lack of standardisation has largely limited the commercial development and application of polyphenol–polysaccharide complex. Given these limitations, it is crucial to establish robust qualitative and quantitative assays for polyphenol–polysaccharide complex, as well as their pharmacokinetics and tissue distribution, in biological samples such as blood, tissues, and excreta from mice.

In this study, the *Hizikia fusiformis* polyphenol–polysaccharide complex and its purified fractions were synthesised as fluorescently labelled products of the *Hizikia fusiformis* polyphenol–polysaccharide complex by connecting them with FITC through Tyr as a linker arm. By exploring the feasibility of the method, the pharmacokinetics and tissue distribution of polyphenol–polysaccharide complexes and their purified fractions in plasma were studied. This study successfully clarified the pharmacokinetics of the polyphenol–polysaccharide complex in vivo, addressing a gap in the research on the distribution of natural polyphenol–polysaccharide complex in vivo. This method not only promotes the development of pharmacological activities of natural polyphenol–polysaccharide complex but also provides a reference for the detection and analysis of other polyphenol–polysaccharide complexes with bioactive substances.

## 2. Materials and Methods

### 2.1. Materials

*Hizikia fusiforme* was purchased from Dongtou, Zhejiang Province, stored in the National Seaweed Processing Research and Development Technology Sub-centre (Dalian, China) and identified as *Hizikia fusiforme* by Prof. Zhang Zeyu, an algae expert from Dalian Ocean University (No. DLOU-24.03.15). FITC (purity > 99.0%) was purchased from Sigma Corporation (St. Louis, MO, USA). Sodium bicarbonate, sodium dihydrogen phosphate, disodium hydrogen phosphate, and sodium chloride were purchased from Shanghai Sinopharm Chemical Reagent Co Ltd. (Shanghai, China). Anhydrous ethanol was purchased from Jiangsu Wuxi Yasheng Chemical Co Ltd. (Wuxi, China). Tyramine was purchased from Aladdin Holding Group Limited (Shanghai, China). All other chemicals were of analytical grade and above.

### 2.2. Preparation and Purification of HPC

According to our previous study [13] with slight modifications, the *Hizikia fusiforme* polyphenol–polysaccharide complex was extracted using an enzymatic method combined with an alkaline method. The supernatant obtained from the enzymatic and alkaline extraction was combined, and ethanol was added to a final concentration of 70%. Centrifugation was carried out to obtain the precipitate, which was lyophilised to give the crude polyphenol–polysaccharide complex named as HPC. HPC was made into a 15.0 mg mL^−1^ solution and uploaded onto a DEAE-Sepharose Fast Flow weak anion exchange column (1.6 × 30 cm). Gradient elution with 2.5 M NaCl solution and phosphate buffer at a flow rate of 1.0 mL min^−1^ was performed, and an automatic collector was used to collect the eluate to obtain the purified HPC. The total carbohydrate content was monitored by the phenol-sulphuric acid method [27], the total phenol content by the forintol method [28], and the protein content by the Caulobacter Brilliant Blue method [29]. The purified fractions PC1 and PC4 were finally selected for subsequent studies.

### 2.3. Preparation of FITC Labelled HPC and Its Purified Fractions

For the FITC fluorescent labelling, we referred to the method of Dong et al. [30], with a small modification: 0.4 g HPC, PC1, and PC4 were added to 15 mL of 0.2 mol/L phosphate buffer (pH = 8), 0.4 g of Tyr and 0.15 g of sodium cyanoborohydride, and the reaction was carried out on a shaking table (Longyue Instrument Co., Ltd. Shanghai, China) at 37 °C for 96 h. The supernatant was collected by centrifugation at 37 °C for 96 h, 10,000 r/min; 4 vol of ethanol was added, then centrifuged, and the precipitates were lyophilised, i.e., T-HPC, T-PC1, and T-PC4. 0.4 g of T-HPC, T-PC1, and T-PC4 was dissolved in 20 mL of 0.5 mol/L sodium bicarbonate solution, and 0.4 g of FITC was added and reacted in the dark for 24 h. After centrifugation, the supernatant was collected, 4 vol of ethanol was added, then centrifugation and lyophilisation of the precipitates took place: HPC-FITC, PC1-FITC, PC4-FITC.

### 2.4. Chemical Composition Analysis before and after FITC Labelling

In order to understand the effect of fluorescent labelling on polyphenol–polysaccharide complex, we determined the chemical composition of the samples.

#### 2.4.1. Chemical Composition Analyses

The total carbohydrate content, total phenol content, and protein content of the samples before and after labelling were determined according to the method in Section 2.2.

#### 2.4.2. Monophenols, Monosaccharide Composition, and Molecular Weights

The molecular weight distribution was detected by high-performance gel-permeation chromatography (HPGPC) using a Shimadzu RID-20A oscillometric refractive detector (Shimadzu Corporation, Tokyo, Japan). The gel chromatography column was equipped with a TSK-gel G5000PWxl (7.5 mm × 30.0 cm, TOSOH Co., Ltd., Tokyo, Japan) at a flow rate of 0.5 mL/min, and the injection volume was 20 μL (before and after FITC-labelled samples), and the retention times of the peaks were determined using dextran glycosides with different molecular weights as the standards [31].

The monosaccharide content was determined by high performance liquid chromatography (HPLC) [32]. Briefly, the samples before and after FITC labelling were acid-hydrolysed with trifluoroacetic acid (TFA) solution at 110 °C for 8 h. Subsequently, 1-phenyl-3-methyl-5-pyrazolone (PMP) was added to the acid hydrolysate and derivatised at 70 °C for 40 min, followed by the addition of chloroform to remove residual PMP derivatisation. Finally, the monosaccharide composition of each group of samples was analysed on an Agilent ZORBAX Eclipse XDB C-18 column (4.6 × 250 mm, 2.7 μm, Agilent, Santa Clara, CA, USA).

The monophenol composition referred to the method of Ahilya et al. with slight modifications [33]. Briefly, methanol–water–acetic acid (30:69:1) was added to the samples before and after FITC labelling, vortexed for 2 min, left to stand for 2 min, and the samples were then subjected to a water bath at 70 °C for 50 min, and the supernatant was obtained by centrifugation over a 0.22 μm membrane. An Agilent ZORBAX Eclipse XDB C-18 column (4.6 × 250 mm, 2.7 μm, Agilent, Santa Clara, CA, USA) was used. The monophenol composition of each group of samples was analysed according to the retention time of the standards.

### 2.5. Determination of HPC-FITC, PC1-FITC, and PC4-FITC Substitution Degree

FITC was used as the standard, and the concentration of 0.25 μg/mL was prepared. Amounts of 0, 0.1, 0.2, 0.3, 0.4, 0.5, 0.6, and 0.7 mL of the standard solution of 0.25 μg/mL of FITC were pipetted into a brown PC tube, and then added with deionised water to make up to 5 mL, and the fluorescence intensity of each component was measured at λex = 490 nm and λem = 530 nm with fluorescence iconometer (Varioskan LUX, Thermo Fisher Scientific, Beijing, China), with the reference to the method of Yan et al. [34].

### 2.6. Fourier Transform Infrared Spectroscopy Analysis

FT-IR was used to determine the infrared spectra of HPC, PC1, and PC4 and the three sets of samples after FITC labelling [32]. Spectra were collected at a resolution of 2 cm^−1^ for wave numbers ranging from 400 to 4000 cm^−1^.

### 2.7. Animal Experiments

**Figure 1:**
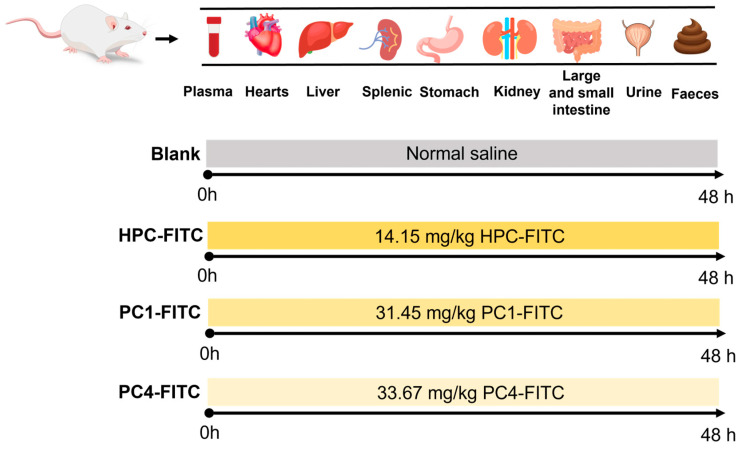
Animal feeding cycle.

ICR mice (SPF grade, 30 ± 4 g, male) were purchased from Liaoning Changsheng Biotechnology Co., Ltd. (Shenyang, China) under the Certificate of Conformity No. SCXK-Liao-2023-074, and the animal experiments were approved by the Animal Ethics Committee of the Ocean University of Dalian (No. DLOU-20231116). The animals were housed in a 12 h circadian light cycle, after seven days of acclimatisation, as shown in Figure 1 and were divided into four groups according to the gavage samples: blank (normal saline); HPC-FITC (14.15 mg/kg); PC1-FITC (31.45 mg/kg); and PC4-FITC (33.67 mg/kg). All mice were fasted before the experiment, but were allowed to drink freely. Samples were given 48 h before execution, and after gavage, five mice were randomly selected at different time points (0.5, 1, 2, 4, 6, 8, 12, 24, 48 h) (Figure 1). Blood was collected from the orbits and then the spine was dislocated and killed, and blood, heart, liver, spleen, stomach, kidney, small intestine, large intestine urine, and faeces were collected for subsequent analysis. Tissue samples were rinsed with saline using absorbent paper to dry the surface water and weighed, added with appropriate amount of PBS solution (pH = 7.2) to prepare as 10% tissue homogenate, and the fluorescence intensity of polyphenol–polysaccharide complex in plasma and tissues was determined at different time points. They were cared for in accordance with the National Research Council’s Guide for the Care and Use of Laboratory Animals.

### 2.8. Development of a Method for the Quantitative Analysis of FITC by HPC and Its Purified Fractions

#### 2.8.1. Establishment of Quantitative Analytical Methods and Sample Preparation

Appropriate amounts of HPC-FITC, PC1-FITC, and PC4-FITC were weighed and prepared into a 1 mg/mL standard solution with PBS buffer, and the standard solution was diluted with PBS buffer to form the standard solution with the following concentrations: 1, 2, 4, 8, 10, 20, 30, 40, and 50 μg/mL.

Amounts of 100 μg/mL of HPC-FITC, PC1-FITC, and PC4-FITC standard solutions were added to the plasma of the blank group, 10% tissue and excreta homogenate, and diluted to three concentrations of 0.5 μg/mL, 5 μg/mL, and 25 μg/mL.

#### 2.8.2. Determination of Sample Recovery, Precision, and Stability

The recovery rate was calculated by monitoring the fluorescence intensity of the fluorescently labelled polyphenol–polysaccharide complex solution containing the three concentrations at five different times of the day. The recovery rate was actual concentration/theoretical concentration × 100.

Precision was determined by monitoring each group of samples for five working days and calculating the relative standard deviation (RSD, %) between intra-day and inter-day periods to test the precision of the method.

The stability of the samples was calculated by measuring the fluorescence intensity of low-, medium- and high-concentration samples under different conditions: room temperature (48 h); −20 °C (15 days); repeated freezing and thawing (3 times).

### 2.9. Establishment of Standard Curves for Plasma and Homogenised Tissue, and Excreta

The standard solutions of plasma, homogenised tissues, and excreta were prepared at concentrations of 0.5, 1, 2.5, 5, 10, 20, and 25 µg/mL, and the fluorescence intensity was measured by aspirating 200 µL of the samples at each concentration, and the standard curves of the mass of polyphenol–polysaccharide complex versus the fluorescence intensity were plotted, and the linear equations were calculated.

### 2.10. Analysis of Plasma Pharmacokinetics

The fluorescence intensity in plasma was measured using the non-atrial model of DAS (Drug and Statistics) 2.0 software (Chinese Pharmacology Society, Shanghai, China), and the pharmacokinetics were calculated in mice after a gavage of HPC-FITC, PC1-FITC, and PC4-FITC. The pharmacokinetic parameters of HPC-FITC, PC1-FITC, and PC4-FITC were calculated as the ratio of maximum blood concentration (C_max_), time to reach maximum blood concentration (T_max_), elimination half-life (T_1/2_), area under the concentration-time curve (AUC), mean residence time (MRT), ratio of plasma clearance (CL), and bioavailability (F).

### 2.11. Determination of Tissue and Faecal Samples

The fluorescence intensity of the tissue and excreta homogenate samples was measured and brought into the standard curve established in Section 2.9; the concentrations of the three groups of samples were calculated in the mouse tissue and excreta, respectively, and the cumulative excretion in mice and the cumulative excretion as a percentage of the administered dose at 48 h were calculated separately.

### 2.12. Statistical Analyses

Each experiment had at least three parallel data and the results were expressed as mean ± SD. Data were processed and statistically analysed using DAS 2.0 and SPSS 26.0 software. *p* < 0.05 indicates significant differences. All figures were produced by Origin 2021 and Figdraw 2.0.

## 3. Results and Discussion

### 3.1. Chemical Composition Analysis of HPC and Purified Fractions before and after FITC Labelling

**Figure 2:**
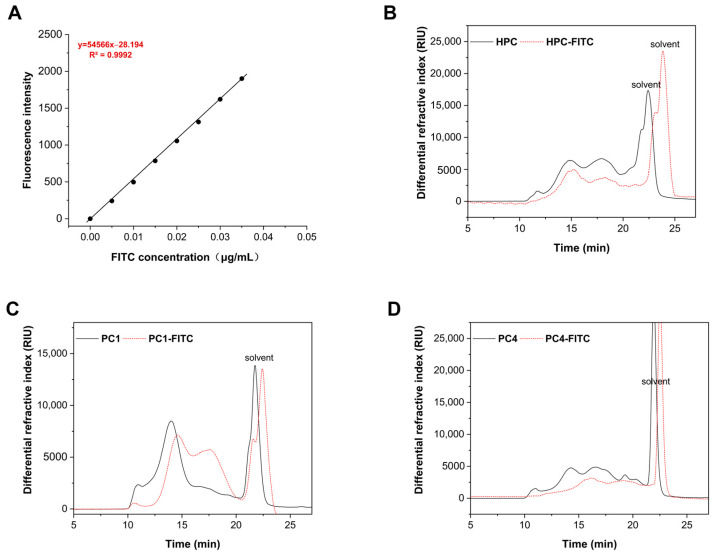
The regression equation curve of FITC (**A**) and HPGPC spectra of samples and FITC-labelled samples: (**B**) HPC; (**C**) PC1; (**D**) PC4.

The total carbohydrate content, total phenol content, protein content, and molecular weight of HPC (68.32 ± 0.28%, 59.43 ± 0.07 mg GAE/g, 3.17 ± 0.19%), PC1 (45.61 ± 0.16%, 18.42 ± 0.03 mg GAE/g, 1.13 ± 0.09%), and PC4 (50.43 ± 0.25%, 40.33 ± 0.21 mg GAE/g, 1.43 ± 0.43%) are shown in Table 1. After the fluorescence labelling reaction, both the total carbohydrate content (60.81 ± 0.21%, 38.52 ± 0.37%, 43.67 ± 0.17%), total phenol content (49.28 ± 0.18 mg GAE/g, 12.96 ± 0.10 mg GAE/g, 35.71 ± 0.07 mg GAE/g), and protein content (1.98 ± 0.03%, 0.46 ± 0.01%, 0.93 ± 0.01%) showed a slight decrease compared to the unlabelled samples, which could be attributed to the alkaline conditions used during the labelling process. The monosaccharide composition analysis revealed that the primary monosaccharides in HPC, PC1, and PC4 were Man, Glu, Gal, and Fuc, and the composition remained nearly unchanged after labelling, indicating that the labelling process did not significantly affect the monosaccharide composition of the polyphenol–polysaccharide complex. This observation is consistent with the findings of Dong et al. [30]. Similarly, the monophenol composition before and after labelling showed negligible variation. The molecular weights (Mws) of the polyphenol–polysaccharide complexes and their purified fractions were HPC: 346.8 kDa, PC1: 328.0 kDa, PC4:129.3 kDa; the Mws after FITC labelling were HPC-FITC: 337.6 kDa, PC1: 319.8 kDa, PC4: 121.4 kDa. This indicated that the retention time of the three sample groups during the assay process was nearly identical before and after labelling, although there was a slight reduction in molecular weight after fluorescent labelling (Figure 2B–D). This result is in line with the findings of Ting et al. [35]. Collectively, these findings demonstrate that FITC-labelled polyphenol–polysaccharide complexes maintain their structural integrity with minimal impact on their molecular framework. Thus, the results indicate that fluorescent labelling does not significantly alter the physicochemical properties and chemical composition of the samples.

### 3.2. Validation of Fluorescent Labelling

#### 3.2.1. Degree of Substitution

**Figure 3:**
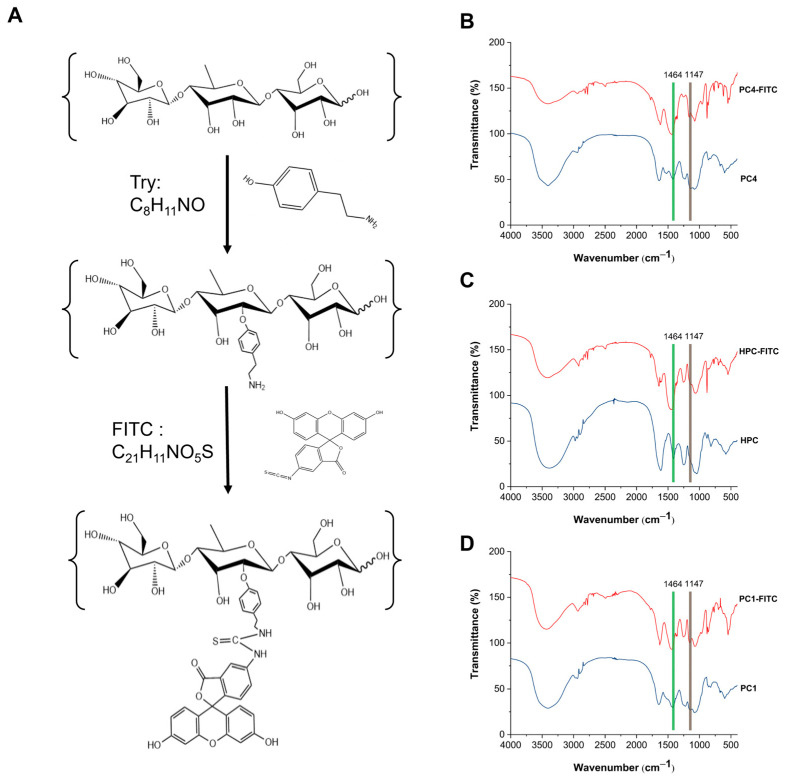
Fluorescence labelling process (**A**) of FITC and Fourier transform infrared spectroscopy of HPC-FITC (**B**), PC1-FITC (**C**), PC4-FITC (**D**).

As shown in Figure 2A, the FITC standard curve (demonstrated the regression equation: y = 54566x − 28.194 (R^2^ = 0.9992)), indicated excellent linearity. By substituting the measured fluorescence intensities of HPC-FITC, PC1-FITC, and PC4-FITC into this standard curve, the average substitution degrees of FITC in HPC, PC1, and PC4 were found to be 0.471%, 0.212%, and 0.198%, respectively. Notably, the fluorescence substitution rate of HPC-FITC was significantly higher than that of PC1-FITC and PC4-FITC. This difference may be attributed to the smaller molecular weights of PC1 and PC4 and their fewer reducing ends. During the repeated alcohol precipitation process used to obtain fluorescent markers, the incomplete precipitation of labelled PC1-FITC and PC4-FITC likely led to some loss of these fractions [36]. The above results indicate that FITC was successfully labelled on the polyphenol–polysaccharide complex. However, it remains unclear whether the FITC labelling has any effect on the structure of the polyphenol–polysaccharide complex, which still requires further investigation. The synthesis process and chemical structures are illustrated in Figure 3A.

#### 3.2.2. Fourier Transform Infrared Spectroscopy

Figure 3B–D shows the FT-IR spectra of HPC-FITC, PC1-FITC, and PC4-FITC. The absorption intensities of the functional groups in HPC-FITC, PC1-FITC, and PC4-FITC differ, the intense and broad absorption peak at 3400 cm^−1^ is associated with the tensile vibration of O-H. The feeble absorption peak at 2950 cm^−1^ might be attributed to C-H tensile vibration. The stretching vibration peaks of C=O and C=O=C were detected at 1625 cm^−1^ and 1250 cm^−1^, and the characteristic peaks of sugar appeared at all the above four points. The symmetrical stretching vibration peak of pyranose was witnessed at 1060 cm^−1^. At 882 cm^−1^; this indicates that sulfate is attached to the C4 position of fucose, and that the fluorescence labelling reaction did not affect the sugar backbone [13,32,37]. The vibrational peaks of the C-S bond at around 1160 cm^−1^ and the characteristic absorption peaks of aromatics at around 1^−1^¹ [36] confirm that FITC was successfully attached to the sugar chain. This suggests that the labelling process did not alter the sugar chain structure, which is consistent with the results of the previous monosaccharide compositions. These findings indicate that the polyphenol–polysaccharide complexes were successfully labelled, establishing a basis for subsequent in vivo experiments.

### 3.3. Establishment and Validation of Quantitative Analysis Methods

#### 3.3.1. Standard Curve Plotting

According to the standard curve, we measured the fluorescence intensity in heart, liver, spleen, stomach, kidney, large intestine, small intestine, faeces, urine and plasma after adding three groups of samples. The correlation coefficients of the linear regression equations for each biological tissue sample were all greater than 0.99 (Table 2), demonstrating good linearity in the range of 0.5–25 μg/mL. This indicates that the linear equations meet the requirements for pharmacokinetic studies.

#### 3.3.2. Recovery, Precision, and Stability

The recoveries, precision, and stability of the quantitative analysis methods for HPC-FITC, PC1-FITC, and PC4-FITC were evaluated. The results showed that the recoveries of each group of labelled complexes ranged from 93.19% to 106.54% (Tables S1–S3). The recoveries of the labelled polyphenol–polysaccharide complex were all above 90%, demonstrating that this method is suitable for analysing biological samples. The accuracy, intra-day precision, and inter-day precision of FITC in different samples ranged from 1.38% to 7.38%, 1.09% to 6.33%, and 1.37% to 6.83%, respectively (Table S4), all of which were less than 15%. Thus, the method precision of this experimental design meets the requirements for determining biological samples.

The stability of the biological samples was verified by testing them after 48 h of standing at room temperature, 15 days of storage at −20 °C, and three cycles of freeze-thawing. Under these conditions, the RSD values of the sample concentrations in plasma, heart, liver, spleen, kidney, stomach, small intestine, large intestine, urine, faeces, and other tissues of mice were less than 15% (Tables S5–S7). These values were within the acceptable range, indicating that the samples were stable and met the stability requirements.

In summary, the results of these experiments demonstrate the stability, precision, and accuracy of the methods used, fulfilling the basic criteria for conducting pharmacokinetic studies.

### 3.4. In Vivo Pharmacokinetic and Tissue Distribution Studies of Labelled Polyphenol–Polysaccharide Complex

#### 3.4.1. Pharmacokinetic Parameters of HPC-FITC, PC1-FITC, PC4-FITC in Mice by Gavage

**Figure 4:**
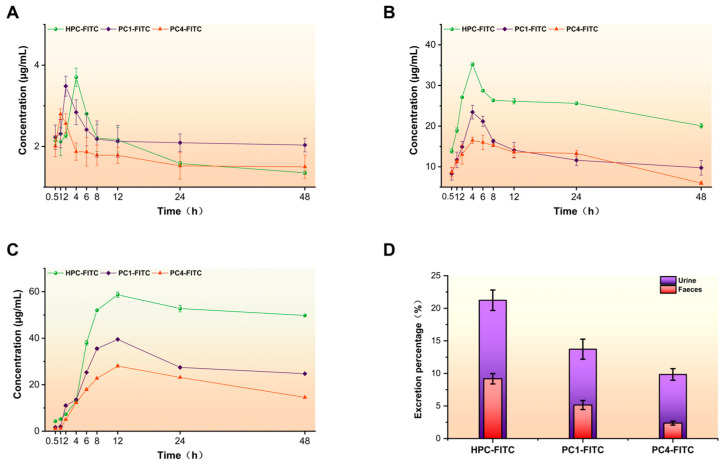
Oral HPC in mice—FITC, PC1—FITC, and PC4—FITC within 48 h of blood drug concentration (**A**); cumulative excretion of urine (**B**) and faeces (**C**); cumulative excretion of urine and faeces (**D**) over 48 h as a percentage of the dose administered.

Pharmacokinetics and tissue distribution are key to understanding biological activity. The results of these experiments demonstrated that HPC-FITC, PC1-FITC, and PC4-FITC exhibit good stability. To further understand their absorption and metabolic properties in vivo, we monitored the blood concentration levels and tissue distribution in mice for 48 h after administration. Pharmacokinetic parameters were calculated for the plasma concentrations of HPC-FITC, PC1-FITC, and PC4-FITC after gavage. Figure 4A shows the average blood concentrations of the three groups of samples, and the corresponding pharmacokinetic parameters are presented in Table 3. The results showed that the absorption rates of PC1-FITC and PC4-FITC were significantly higher than that of HPC-FITC, with maximum peak concentrations (C_max_) of 3.71, 3.48, and 2.79 µg/mL, respectively. Through Table 3 we find that the distribution and elimination of FITC in mice were found to be slower compared to PC1-FITC and PC4-FITC, with a longer half-life. The mean retention times (MRT_0–∞_) in vivo for the three groups of samples were HPC-FITC: 36.48 h; PC1-FITC: 18.06 h; and PC4-FITC: 16.88 h. The time required for elimination of the drug from the body was shorter for PC1-FITC and PC4-FITC compared to HPC-FITC. Polysaccharides with higher molecular weights usually have longer mean retention times (MRT) [38]. In a study by Bi et al., it was found that FITC-labelled *Polygonatum sibiricum* polysaccharides (PRP-TYR-FITC) reached a maximum peak concentration (C_max_) within 2 h of oral administration, followed by a slow clearance from plasma, and had a long half-life (T_1/2_) of 31.39 h [39]. Zhang et al. observed that low-molecular-weight fucoidan (7.1 kDa) disappeared from the bloodstream very quickly after intravenous injection in rabbits (T_1/2_ = 11.24 ± 2.93 min, MRT = 109 min), whereas high-molecular-weight fucoidan administered by gavage prolonged the mean retention time, with an MRT value of 6.79 ± 1.63 h [40]. Wang et al. further demonstrated that the T_1/2_ and MRT_0–∞_ values of PHZ decreased with enzymatic degradation. By comparing the pharmacokinetics of Phlorizin with and without enzymatic treatment, they showed that small molecule drugs are more readily absorbed and utilised by the organism [41]. Combined with the results of these experiments, it is clear that HPC-FITC has poorer gavage absorption and a longer retention time, which may be related to its larger molecular weight.

KANEO et al. conducted a systematic assay of blood concentrations of dextrans at different molecular weights (Mws) in mice and found that dextrans with molecular weights below 20 kDa were rapidly eliminated from the blood and exhibited poor hepatic aggregation (2.1–3.7%). In contrast, when the molecular weight exceeded 40 kDa, dextrans were significantly distributed in the liver (18.9–24.0%) and remained for a longer period. The pharmacokinetic differences between dextrans with Mws ≤ 20 kDa and Mws ≥ 40 kDa were significant, which may be related to the pore size of the renal vascular bed. Additionally, changes in the renal clearance of dextrans with different Mws were more pronounced than those in hepatic clearance, possibly due to differences in the structure of renal and hepatic capillary walls [42]. Woting et al. determined the relationship between small intestinal permeability, intestinal transit time, and the molecular weight of FITC dextrans in C3H mice, using low-molecular-weight (4 kDa) and high-molecular-weight (70 kDa) FITC dextrans. Their findings revealed a clear relationship between molecular weight, intestinal transit time, and the uptake of dextrans in these mice. The results showed that 4 kDa FITC dextran was preferentially absorbed in the duodenum and jejunum. As the plasma concentration of 4 kDa FITC dextran began to decrease, 70 kDa FITC dextran reached the ileum in all mice [43]. Zheng et al. found that the effect of molecular weight on the pharmacokinetics of polysaccharides is determined by two factors. Firstly, small-molecular-weight polysaccharides are rapidly excreted through the kidneys. In contrast, large-molecular-weight polysaccharides reduce renal clearance, causing them to remain in the circulatory system for a longer period and facilitating their aggregation in the liver. Secondly, a further increase in molecular weight decreases the permeability of polysaccharides into hepatocytes, thereby reducing hepatic accumulation [44].

In summary, we find that natural polysaccharides possess a complex structure, a large molecular weight, and a longer metabolic process in contrast to small-molecule drugs. Meanwhile, small-molecule substances have a higher absorption rate within the body and are more readily absorbed and utilised by it, whereas large-molecule substances are digested and absorbed at a slower pace within the body, mainly through the intestinal–brain microbial axis, which can improve gut microbiota and potentially slow down disease progression [45,46,47,48], which is consistent with our findings. These results indicate that the FITC method is stable, rapid, and sensitive, and is suitable for the pharmacokinetic study of the polyphenol–polysaccharide complex of *Hizikia fusiforme*.

#### 3.4.2. Excretion Ratios of Gavage HPC-FITC, PC1-FITC, and PC4-FITC in Mice

The results of metabolic experiments were used to calculate the cumulative excretion and the cumulative excretion as a percentage of the administered dose at 48 h. As shown in Figure 4B–D, after gavage of HPC-FITC, PC1-FITC, and PC4-FITC, the primary time period for urinary excretion was 0–6 h. Urinary excretion peaked at 4 h (HPC-FITC: 35.18 ± 0.41%, PC1-FITC: 23.44 ± 1.68%, PC4-FITC: 16.49 ± 0.81%), and then the excretion rate gradually decreased with time. The excretion rate in faeces did not change significantly from 0 to 6 h. After 6 h, the faecal excretion rate gradually increased and reached a peak at 12 h (HPC-FITC: 58.71 ± 1.15%, PC1-FITC: 39.50 ± 0.35%, PC4-FITC: 28.01 ± 0.51%). The cumulative excretion rates of HPC-FITC, PC1-FITC, and PC4-FITC in urine as a percentage of the total dose were 9.18%, 2.37%, and 13.72%, respectively, and the cumulative excretion rates in faeces as a percentage of the total dose were 5.15%, 21.23%, and 9.84%, after 48 h of administration by gavage in mice. These results suggest that the retention of polyphenol–polysaccharide complex in the intestine may contribute to their potential effects on the gut [13,45]. The main metabolic pathway of the three samples was excretion via urine and faeces through the kidneys and intestines. Chao et al. found that the concentration of labelled *Lycium barbarum* polysaccharide (LBP) was significantly reduced in all tissues after 24 h of drug administration, but the concentration of LBP-FITC in the large intestine, liver, and kidneys was slightly higher. This suggests that LBP-FITC is mainly excreted in faeces in addition to urine via the kidneys, which is similar to our findings [49]. These metabolic kinetic parameters indicate that the molecular weights of HPC-FITC, PC1-FITC, and PC4-FITC directly influence the rate of drug absorption and elimination time after gavage in mice. The faster absorption of smaller polyphenol–polysaccharide complex aligns with previous pharmacokinetic studies. Similar results were observed in pharmacokinetic studies of labelled *Glycyrrhiza uralensis* polysaccharides (GUPS) in rats by Abudukahaer et al., and natural polyphenols like quercetin by Gaber et al., which also concluded that smaller molecules have a faster rate of absorption [50,51].

#### 3.4.3. Tissue Distribution Pattern of HPC-FITC, PC1-FITC, and PC4-FITC in Mice by Gavage

**Figure 5:**
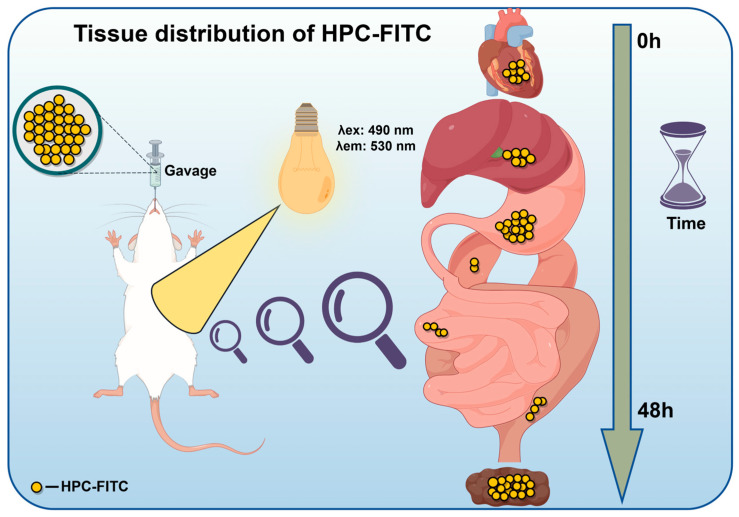
Tissue distribution of FITC-labelled *Hizikia fusiforme* polyphenol–polysaccharide complex in mice from 0–48 h (yellow dots represent HPC).

The organisational distribution of HPC-FITC, PC1-FITC, and PC4-FITC is depicted in Figure 5, and the corresponding parameters are presented in Table 4. The results indicated that HPC-FITC, PC1-FITC, and PC4-FITC were least distributed in the heart and spleen, with the highest distribution observed in the stomach and intestines, followed by the kidneys and liver. After 0.5 h of gavage administration, HPC-FITC (23.64 ± 0.06 μg/mL), PC1-FITC (22.53 ± 0.02 μg/mL), and PC4-FITC (9.98 ± 0.05 μg/mL) were detected in the stomach at the highest concentrations, which then declined rapidly. In the small intestine, the concentrations of these complexes peaked at 2–4 h and subsequently decreased. In the large intestine, concentrations gradually increased from 2 h, peaking at 6 h before declining. Fewer concentrations of HPC-FITC, PC1-FITC, and PC4-FITC were found in the liver and kidneys; however, the concentrations in the kidneys increased significantly after 2 h, reaching 13.35 ± 0.03 μg/mL, 8.37 ± 0.12 μg/mL, and 4.36 ± 0.08 μg/mL, respectively, by 4 h. This suggests that the kidneys have a strong capacity for polyphenol–polysaccharide complex uptake. These results indicate that HPC-FITC, PC1-FITC, and PC4-FITC are absorbed into the blood and subsequently excreted in urine through the kidneys after 2 h of gavage, which aligns with findings by Wang et al. and Zhang et al. [52,53]. Additionally, Xu et al. found a similar strong uptake capacity for FITC-labelled fucoidan in the kidneys [21].

Among the samples, PC1 and PC4 showed lower response values and shorter retention times compared to HPC. The significant differences in tissue distribution may be related to the speed of uptake by the organism, molecular weight, or the size of vascular apertures in the kidneys and liver [54,55,56]. Dou et al. studied the absorption and distribution of *Millettia speciosa* polysaccharide (MSP) labelled with a Rhodamine B fluorescent marker in mice. Their results showed that MSP-Rh B was mainly distributed in the gastrointestinal tract, liver, and kidneys. Following intragastric administration, it first entered the systemic circulation and then distributed to the liver and kidneys, with fluorescent signals gradually weakening over time, which is consistent with our findings [57]. Several studies have confirmed that hepatic accumulation and renal excretion of active substances are influenced by molecular weight. Bi et al. observed fluorescence in rat glomerular filtration membranes and suggested that macromolecule polysaccharides may be sequestered by these membranes [39]. Song et al. found that FITC-labelled mannuronic acid (FITC-PM; 16.83 kDa) was predominantly distributed in the kidneys (maximum concentration at 0.5 h), followed by the liver, and high concentrations of PM were present in the kidneys even when undetectable in the blood [58]. Of course, our study only analysed the distribution pattern of ICR mice, which has some limitations. Future studies should investigate the metabolic pattern of the *Hizikia fusiforme* polyphenol–polysaccharide complex in different animal models. In summary, PC1 and PC4 are absorbed into the bloodstream and distributed more rapidly compared to the HPC purified group.

## 4. Conclusions

In this study, we used FITC to label HPC and its purified fractions, creating isothiocyanine fluorescein-labelled polyphenol–polysaccharide complex. Chemical composition analysis confirmed that this labelling approach did not affect the structure of the polyphenol–polysaccharide complex. The quantitative analysis of HPC-FITC, PC1-FITC, and PC4-FITC in vivo was successfully established, demonstrating the feasibility of using FITC for labelling polyphenol–polysaccharide complex. This method was also applied to investigate the pharmacokinetics and tissue distribution of the three sample groups in mice. And the animals in this experiment were treated humanely to minimise animal injuries, respect animal rights, and protect animal safety. The pharmacokinetic results from a single oral administration revealed that HPC-FITC had a longer half-life and slower elimination compared to the purified fractions, indicating that polyphenol–polysaccharide complexes with smaller molecular weights are absorbed more rapidly. Additionally, we found that the three sample groups were primarily distributed in the stomach, small intestine, large intestine, kidneys, and liver. The notable distribution in the kidneys and liver suggests that these organs have a significant uptake capacity for the drug. Excretion studies showed that most of the samples from all three groups were excreted through the urine and faeces. Moreover, after 48 h of administration by gavage, the cumulative excretion of HPC-FITC accounted for 5.15% of the total administration dose, which proved that the macromolecular polyphenol–polysaccharide complex could play its potential role in the intestine due to its difficult absorption. In summary, this study provides insights into the mechanism of energy expansion and potentiation of polyphenol–polysaccharide complex, elucidating their digestion and absorption patterns in vivo. This research offers a theoretical foundation for developing algal-based natural functional foods.

## Figures and Tables

**Table 1:** Chemical composition of the sample.

	HPC	PC1	PC4	HPC-FITC	PC1-FITC	PC4-FITC
Total carbohydrates (%, *w*/*w*)	68.32 ± 0.28 ^a^	45.61 ± 0.16 ^a^	50.43 ± 0.25 ^a^	60.81 ± 0.21 ^b^	38.52 ± 0.37 ^b^	43.67 ± 0.17 ^b^
Total phenols (mg GAE/g)	59.43 ± 0.07 ^a^	18.42 ± 0.03 ^a^	40.33 ± 0.21 ^a^	49.28 ± 0.18 ^b^	12.96 ± 0.10 ^b^	35.71 ± 0.07 ^b^
Protein content (%)	3.17 ± 0.19 ^a^	1.13 ± 0.09 ^a^	1.43 ± 0.43 ^a^	1.98 ± 0.03 ^b^	0.46 ± 0.01 ^b^	0.93 ± 0.01 ^b^
Molecular weight (kDa)	346.8	328.0	129.3	337.6	319.8	121.4
Monosaccharide composition (%)						
Man	14.58	11.97	10.30	13.47	12.01	10.68
Rha	1.40	1.79	2.91	1.43	1.81	2.37
GluA	0.45	0.21	5.25	0.77	0.18	4.86
GalA	0.94	0.40	2.06	1.06	0.45	2.94
Glu	9.91	1.14	5.99	9.76	1.37	5.14
Gal	24.60	25.53	15.71	24.77	25.84	14.28
Xyl	2.75	2.05	1.91	2.61	1.68	2.03
Fuc	45.35	56.87	55.83	46.12	56.65	57.69
Monophenol composition (%)						
GA	59.30	22.75	37.07	58.96	23.16	36.28
EGC	24.66	68.32	47.76	24.18	68.47	48.11
EC	--	4.03	--	--	3.65	--
ECG	12.32	4.89	--	13.28	4..27	--
CG	3.8	--	15.16	3.57	--	15.60

Note: A different superscript letter on the same line indicates a significant difference (*p* < 0.05); Man, mannose; Rha, rhamnose; GluA, glucuronic acid; GlaA, galactose acid; Glu, glucose; Gal, galactose; Xyl, xylose; Fuc, fucose; GA, gallic acid; EGC, (-) epigallocatechin; C, catechin hydrate; EC, epicatechin; ECG, (-) epicatechin gallate; CG, catechin gallate.

**Table 2:** The linear regression equation and the correlation coefficients of biological samples.

	HPC-FITC		PC1-FITC		PC4-FITC	
Heart	y = 1525.7x − 551.44	R^2^ = 0.9992	y = 1063.9x − 263.73	R^2^ = 0.9991	y = 1464.6x − 175.6	R^2^ = 0.9995
Liver	y = 1474.2x − 462.06	R^2^ = 0.9990	y = 981x − 44.61	R^2^ = 0.9998	y = 1486.7x − 202.96	R^2^ = 0.9998
Splenic	y = 1526.7x − 321.98	R^2^ = 0.9994	y = 1090x − 155.77	R^2^ = 0.9990	y = 1559.3x − 244.09	R^2^ = 0.9994
Stomach	y = 1477.4x − 183.7	R^2^ = 0.9964	y = 1058x − 76.085	R^2^ = 0.9995	y = 1468.9x − 163.09	R^2^ = 0.9994
Kidney	y = 1609.8x − 578.4	R^2^ = 0.9993	y = 1048.6x − 59.47	R^2^ = 0.9994	y = 1533.6x − 190.5	R^2^ = 0.9986
Large intestine	y = 1700.6x − 106.23	R^2^ = 0.9993	y = 1251.2x − 178.64	R^2^ = 0.9955	y = 1699.7x − 173.27	R^2^ = 0.9996
Small intestine	y = 1866.8x − 242.15	R^2^ = 0.9994	y = 1388.1x − 378.35	R^2^ = 0.9979	y = 1888x + 136.49	R^2^ = 0.9999
Faeces	y = 2347.6x − 372.51	R^2^ = 0.9996	y = 1609x − 242.69	R^2^ = 0.9995	y = 2231.7x − 42.249	R^2^ = 0.9988
Urine	y = 2411.8x + 1003.9	R^2^ = 0.9995	y = 1563.3x + 936.52	R^2^ = 0.9998	y = 2353.1x + 444.9	R^2^ = 0.9999
Plasma	y = 1332x − 155.09	R^2^ = 0.9991	y = 1025.9x − 43.727	R^2^ = 0.9989	y = 1440.8x − 75.757	R^2^ = 0.9992

**Table 3:** Pharmacokinetic parameters of HPC-FITC, PC1-FITC, and PC4-FITC after oral administration.

Pharmacokinetic Parameters	Result ± SD		
	HPC-FITC	PC1-FITC	PC4-FITC
C_max_ (mg/mL)	3.71 ± 0.16	3.48 ± 0.18	2.79 ± 0.06
T_max_ (h)	4.00 ± 0.31	2.00 ± 0.01	1.00 ± 0.03
T_1/2_ (h)	26.92 ± 0.76	25.04 ± 1.25	24.12 ± 0.61
AUC_0–t_ (mg/L h)	47.63 ± 1.13	64.28 ± 3.38	147.82 ± 15.62
AUC_0–∞_ (mg/L h)	56.02 ± 3.92	66.37 ± 2.03	169.38 ± 13.38
MRT_0–∞_ (h)	36.48 ± 0.10	18.06 ± 0.21	16.88 ± 0.17
CL/F (L/h/kg)	1.27 ± 0.02	0.76 ± 0.10	0.31 ± 0.04

Notes: T_max_, time to peak concentration; C_max_, peak concentration; T_1/2_, half-life; AUC_0–t_, area under the curve from zero to t; AUC_0–∞_, area under the curve from zero to infinity; MRT_0–∞_, mean residence time; CL/F: ratio of plasma clearance to absolute bioavailability.

**Table 4:** Tissue distribution of HPC-FITC, PC1-FITC, and PC4-FITC in mice orally (n = 5).

Tiusse	Group	Time (h)								
		0.5	1	2	4	6	8	12	24	48
Heart	HPC-FITC	2.27 ± 0.03	2.59 ± 0.07	2.85 ± 0.02	2.86 ± 0.04	2.83 ± 0.02	2.68 ± 0.05	2.59 ± 0.01	1.52 ± 0.03	1.24 ± 0.03
	PC1-FITC	3.01 ± 0.04	3.60 ± 0.08	3.53 ± 0.06	3.30 ± 0.05	3.05 ± 0.08	2.88 ± 0.02	2.58 ± 0.09	2.71 ± 0.02	1.66 ± 0.05
	PC4-FITC	2.14 ± 0.11	2.17 ± 0.14	2.67 ± 0.16	2.73 ± 0.12	2.63 ± 0.15	2.41 ± 0.09	2.52 ± 0.07	2.21 ± 0.04	1.45 ± 0.03
Liver	HPC-FITC	3.21 ± 0.22	3.18 ± 0.11	3.71 ± 0.05	3.75 ± 0.04	3.35 ± 0.06	2.91 ± 0.05	2.64 ± 0.13	2.30 ± 0.02	1.14 ± 0.04
	PC1-FITC	1.93 ± 0.17	3.36 ± 0.06	4.25 ± 0.12	4.61 ± 0.14	4.39 ± 0.08	3.71 ± 0.13	3.65 ± 0.09	3.54 ± 0.05	1.37 ± 0.12
	PC4-FITC	2.12 ± 0.22	2.54 ± 0.21	2.64 ± 0.12	2.60 ± 0.18	2.48 ± 0.17	2.22 ± 0.04	2.16 ± 0.13	1.41 ± 0.17	0.97 ± 0.12
Splenic	HPC-FITC	0.79 ± 0.02	0.63 ± 0.04	0.77 ± 0.03	0.74 ± 0.11	1.24 ± 0.03	1.11 ± 0.05	0.99 ± 0.04	0.83 ± 0.07	0.61 ± 0.02
	PC1-FITC	0.96 ± 0.06	0.99 ± 0.12	1.15 ± 0.13	1.16 ± 0.11	1.61 ± 0.22	1.57 ± 0.18	1.35 ± 0.12	0.93 ± 0.10	0.74 ± 0.16
	PC4-FITC	0.72 ± 0.06	0.69 ± 0.04	0.82 ± 0.03	0.97 ± 0.11	1.17 ± 0.09	1.13 ± 0.17	0.83 ± 0.08	0.72 ± 0.06	0.59 ± 0.05
Stomach	HPC-FITC	23.64 ± 0.06	23.34 ± 0.05	10.28 ± 0.11	7.55 ± 0.02	6.61 ± 0.04	5.66 ± 0.07	3.40 ± 0.04	2.24 ± 0.13	2.09 ± 0.06
	PC1-FITC	22.53 ± 0.02	7.30 ± 0.06	5.46 ± 0.05	4.54 ± 0.04	3.95 ± 0.08	3.93 ± 0.02	3.84 ± 0.05	2.97 ± 0.01	2.72 ± 0.04
	PC4-FITC	9.98 ± 0.05	8.22 ± 0.04	5.85 ± 0.03	2.50 ± 0.02	2.51 ± 0.16	1.73 ± 0.09	1.72 ± 0.08	1.42 ± 0.06	1.34 ± 0.12
Kidney	HPC-FITC	2.63 ± 0.14	4.02 ± 0.06	5.88 ± 0.15	13.35 ± 0.03	4.30 ± 0.03	4.25 ± 0.15	4.09 ± 0.05	2.49 ± 0.03	1.68 ± 0.14
	PC1-FITC	6.15 ± 0.08	6.97 ± 0.04	7.63 ± 0.17	8.37 ± 0.12	6.28 ± 0.09	6.01 ± 0.08	5.70 ± 0.07	2.89 ± 0.02	2.49 ± 0.10
	PC4-FITC	3.96 ± 0.04	4.38 ± 0.22	4.36 ± 0.04	4.36 ± 0.08	4.35 ± 0.12	4.00 ± 0.05	3.74 ± 0.07	2.58 ± 0.16	1.77 ± 0.07
Large intestine	HPC-FITC	1.83 ± 0.18	4.12 ± 0.06	5.13 ± 0.05	8.61 ± 0.05	10.78 ± 0.1	7.31 ± 0.17	4.42 ± 0.10	1.36 ± 0.14	1.07 ± 0.03
	PC1-FITC	3.45 ± 0.10	4.47 ± 0.02	6.65 ± 0.06	7.8 ± 0.10	9.77 ± 0.05	7.12 ± 0.03	6.24 ± 0.06	3.31 ± 0.13	1.61 ± 0.13
	PC4-FITC	0.79 ± 0.08	1.49 ± 0.13	2.21 ± 0.10	3.61 ± 0.12	4.67 ± 0.06	2.85 ± 0.09	1.67 ± 0.03	1.34 ± 0.04	1.21 ± 0.11
Small intestine	HPC-FITC	2.25 ± 0.09	3.26 ± 0.03	8.78 ± 0.04	14.76 ± 0.13	9.27 ± 0.05	3.73 ± 0.05	2.02 ± 0.11	1.56 ± 0.07	1.28 ± 0.11
	PC1-FITC	2.74 ± 0.07	4.08 ± 0.013	8.91 ± 0.21	5.56 ± 0.03	2.46 ± 0.13	2.37 ± 0.08	2.15 ± 0.19	1.83 ± 0.17	1.87 ± 0.14
	PC4-FITC	2.64 ± 0.05	6.95 ± 0.14	1.59 ± 0.06	1.26 ± 0.03	1.21 ± 0.12	1.19 ± 0.10	1.19 ± 0.06	1.03 ± 0.08	0.99 ± 0.04

## Data Availability

The original contributions presented in the study are included in the article and Supplementary Materials, further inquiries can be directed to the corresponding author.

## Supplementary Materials

The following supporting information can be downloaded at https://www.mdpi.com/article/10.3390/foods13183019/s1, Table S1: Recovery of HPC-FITC in mouse plasma (n = 5); Table S2. Recovery of PC1-FITC in mouse plasma (n = 5); Table S3. Recovery of PC4-FITC in mouse plasma (n = 5); Table S4. Intra-day and inter-day precision of HPC-FITC, PC1-FITC, and PC4-FITC in mouse plasma (n = 5); Table S5. Stability of HPC-FITC in mouse plasma (n = 5); Table S6. Stability of PC1-FITC in mouse plasma (n = 5); Table S7. Stability of PC4-FITC in mouse plasma (n = 5).
