# Supplementary material for: In Vivo Tissue Distribution and Pharmacokinetics of FITC-Labelled Hizikia fusiforme Polyphenol–Polysaccharide Complex in Mice
Source: Foods. 2024 Sep 23;13(18):3019. doi: 10.3390/foods13183019 (PMC11431462; doi:10.3390/foods13183019)
Supplement: Supplementary file 1 [file foods-13-03019-s001.zip › foods-3215991-supplementary.pdf]

Table. S1. Recovery of HPC-FITC in mouse plasma (n=5).

Tissue	Theoretical concentration (μg/mL)	Actual concentration (μg/mL)	Recovery rate (%)	RSD (%)
	0.5	0.48±0.07	96.23±1.32	2.77
Heart	5.0	4.99±0.11	99.84±2.37	5.64
	25.0	25.01±0.29	100.04±1.18	7.33
	0.5	0.46±0.02	98.43±3.66	4.28
Liver	5	5.03±0.10	100.74±2.07	5.35
	25	24.32±0.09	97.31±3.85	1.62
	0.5	0.51±0.01	102.4±2.10	6.38
Splenic	5	4.81±0.05	96.18±1.19	4.21
	25	24.57±0.44	98.27±1.76	3.98
	0.5	0.49±0.11	97.72±2.55	2.77
Stomach	5	5.00±0.05	100.14±1.27	3.64
	25	24.84±0.26	99.35±1.46	5.33
	0.5	0.53±0.02	105.85±3.31	4.28
Kidney	5	4.97±0.06	99.49±1.89	5.97
	25	23.88±0.39	95.51±1.26	8.62
	0.5	0.49±0.16	98.29±2.91	3.38
Large intestine	5	5.12±0.03	102.40±3.03	6.21
	25	23.87±0.10	95.49±4.16	4.32
	0.5	0.53±0.13	105.27±2.76	2.16
Small intestine	5	4.75±0.16	95.06±3.21	1.74
	25	24.56±0.11	98.24±4.23	4.28
	0.5	0.49±0.09	99.38±1.98	3.47
Feces	5	5.06±0.12	101.27±2.47	2.35
	25	24.46±0.38	97.84±1.52	5.17
	0.5	0.52±0.07	104.68±1.46	4.09
Urine	5	4.76±0.17	95.11±3.42	2.91
	25	24.74±0.09	98.96±3.68	3.23
	0.5	0.48±0.14	96.63±2.85	4.33
Plasma	5	5.33±0.10	106.54±2.09	2.62
	25	24.80±0.06	99.18±2.46	3.45

Table. S2. Recovery of PC1-FITC in mouse plasma (n=5).

Tissue	Theoretical concentration (μg/mL)	Actual concentration (μg/mL)	Recovery rate (%)	RSD (%)
	0.5	0.47±0.11	93.47±2.30	7.33
Heart	5.0	4.73±0.06	94.62±1.27	5.35
	25.0	25.12±0.08	100.48±1.66	4.98
	0.5	0.48±0.01	96.12±3.18	6.26
Liver	5	4.91±0.12	98.27±2.46	4.12
	25	25.05±0.08	100.03±3.10	2.86
	0.5	0.49±0.10	99.04±2.18	5.14
Splenic	5	4.72±0.08	94.38±1.74	3.51
	25	24.30±0.35	97.21±1.42	1.37
	0.5	0.46±0.14	93.72±2.28	1.67
Stomach	5	4.97±0.07	99.44±1.47	3.38
	25	24.15±0.31	96.58±1.25	2.07
	0.5	0.52±0.15	104.28±3.09	1.89
Kidney	5	4.82±0.05	96.47±1.16	5.88
	25	24.33±0.20	97.34±1.09	4.67

Large intestine	0.5	0.49±0.10	99.02±2.13	4.21
	5	5.16±0.04	103.16±3.47	1.33
	25	24.32±0.08	97.29±3.16	2.92
Small intestine	0.5	0.50±0.12	100.47±2.04	1.58
	5	4.69±0.06	93.86±1.21	3.03
	25	24.37±0.08	97.48±3.20	5.88
Feces	0.5	0.51±0.14	102.98±2.08	2.96
	5	4.74±0.09	94.77±1.87	4.15
	25	24.53±0.35	98.14±1.41	5.17
Urine	0.5	0.47±0.07	93.19±1.56	4.01
	5	4.87±0.14	97.46±2.82	1.96
	25	25.07±0.10	100.28±4.08	3.41
Plasma	0.5	0.47±0.19	95.38±3.85	2.82
	5	5.20±0.04	104.04±1.69	1.41
	25	24.69±0.05	98.78±2.14	3.68

Table. S3. Recovery of PC4-FITC in mouse plasma (n=5).

Tissue	Theoretical concentration (μg/mL)	Actual concentration (μg/mL)	Recovery rate (%)	RSD (%)
Heart	0.5	0.47±0.20	94.33±4.03	6.33
	5.0	4.93±0.12	98.72±2.56	5.35
	25.0	25.12±0.08	100.48±3.39	3.98
Liver	0.5	0.49±0.08	98.33±1.64	7.21
	5	4.81±0.07	97.01±1.58	3.12
	25	25.08±0.26	100.80±2.31	4.86
Splenic	0.5	0.48±0.05	97.16±1.17	6.74
	5	5.07±0.02	101.38±1.68	3.38
	25	24.85±0.06	99.41±2.60	4.21
Stomach	0.5	0.48±0.15	96.32±1.05	5.68
	5	5.05±0.14	101.00±2.98	3.89
	25	24.56±0.08	98.26±2.63	8.03
Kidney	0.5	0.51±0.17	102.98±3.47	5.54
	5	4.75±0.22	95.17±4.46	3.41
	25	24.76±0.32	99.04±1.82	1.84
Large intestine	0.5	0.49±0.03	97.82±1.06	4.86
	5	5.09±0.16	101.80±1.28	1.61
	25	24.95±0.13	99.79±2.43	3.48
Small intestine	0.5	0.52±0.11	104.49±2.38	6.37
	5	4.91±0.09	98.18±1.33	4.99
	25	25.09±0.33	100.38±2.82	3.68
Feces	0.5	0.47±0.25	94.48±4.41	6.43
	5	4.89±0.20	97.91±4.17	7.99
	25	25.06±0.10	100.24±3.89	5.89
Urine	0.5	0.47±0.08	95.79±1.68	5.51
	5	5.19±0.17	103.08±1.42	3.59
	25	24.60±0.14	98.43±1.99	2.06
Plasma	0.5	0.49±0.13	98.18±2.65	2.10
	5	4.98±0.15	99.64±3.09	4.18
	25	25.44±0.25	101.76±1.83	6.96

Table. S4. Intra-day and inter-day precision of HPC-FITC、PC1-FITCand PC4-FITC in mouse plasma (n=5).

Tissue	Theoretical concentration (μg/mL)	Measured concentration(μg/mL)			RSD (%)					
					Intra-day			Inter-day		
		HPC-FITC	PC1-FITC	PC4-FITC	HPC-FITC	PC1-FITC	PC4-FITC	HPC-FITC	PC1-FITC	PC4-FITC
Heart	0.5	0.452±0.03	0.286±0.02	0.338±0.10	4.32	4.18	4.37	6.33	5.48	6.03
	5.0	4.213±0.16	3.375±0.14	3.383±0.26	2.16	3.37	3.14	5.35	4.16	5.16
	25.0	24.369±0.28	22.264±0.30	23.933±0.41	1.74	1.09	2.23	2.98	2.77	3.07
Liver	0.5	0.491±0.11	0.219±0.15	0.392±0.07	4.28	4.66	4.18	5.26	5.64	5.88
	5	4.684±0.24	3.233±0.38	3.471±0.22	3.47	4.01	3.31	4.12	5.33	4.32
	25	24.224±0.14	22.314±0.41	23.450±0.30	2.35	3.58	2.56	3.86	4.28	3.23
Splenic	0.5	0.415±0.02	0.245±0.05	0.358±0.13	5.17	4.99	4.65	7.14	5.35	5.09
	5	4.912±0.06	3.985±0.11	3.509±0.08	4.09	3.82	3.90	5.51	4.62	4.12
	25	24.252±0.18	22.486±0.28	23.428±0.22	1.91	2.11	2.28	5.37	3.38	3.38
Stomach	0.5	0.457±0.10	0.276±0.14	0.394±0.16	3.23	3.76	4.03	4.68	4.21	5.70
	5	4.643±0.22	4.023±0.56	4.016±0.34	4.33	3.23	3.37	5.03	3.98	4.96
	25	24.643±0.46	22.945±0.33	23.303±0.43	2.62	1.67	2.55	3.41	2.03	3.42
Kidney	0.5	0.509±0.09	0.345±0.02	0.454±0.04	4.45	3.38	4.61	6.47	4.89	5.16
	5	4.831±0.13	3.338±0.15	4.132±0.10	2.11	2.07	3.11	5.52	3.64	4.07
	25	25.002±0.47	23.169±0.22	24.213±0.27	1.64	1.89	1.91	3.32	2.08	2.88
Large intestine	0.5	0.483±0.02	0.295±0.07	0.384±0.13	6.52	5.88	5.47	7.38	6.33	6.37
	5	4.452±0.09	3.894±0.18	4.398±0.20	3.74	4.67	4.18	5.61	5.47	5.29
	25	24.346±0.16	22.685±0.44	24.877±0.39	1.38	2.21	2.46	3.84	3.89	3.68
Small intestine	0.5	0.398±0.07	0.196±0.03	0.263±0.21	5.61	4.33	5.03	6.65	5.16	6.43
	5	4.241±0.23	4.643±0.22	4.331±0.44	3.98	2.92	3.01	4.21	3.74	4.77
	25	24.033±0.10	22.339±0.19	23.804±0.10	2.19	1.58	1.96	3.75	2.85	2.90
Feces	0.5	0.469±0.04	0.265±0.02	0.401±0.11	3.74	3.03	3.41	4.84	4.32	5.05
	5	4.684±0.32	3.884±0.33	3.682±0.27	4.16	3.88	2.82	5.24	3.98	4.78
	25	24.334±0.35	22.387±0.41	24.994±0.43	3.36	2.96	1.37	4.41	3.66	3.36
Urine	0.5	0.423±0.05	0.237±0.06	0.306±0.01	4.81	4.15	3.68	6.87	5.19	5.17
	5	4.618±0.13	4.231±0.25	4.161±0.11	2.62	3.07	3.12	3.91	4.68	4.47
	25	24.164±0.24	22.996±0.37	23.162±0.19	1.57	1.88	2.54	2.77	2.82	3.45
Plasma	0.5	0.447±0.08	0.262±0.11	0.342±0.18	4.54	3.34	4.76	7.12	4.81	6.46
	5	3.983±0.33	4.552±0.25	3.334±0.49	3.36	2.28	3.43	5.38	3.23	4.37
	25	24.128±0.46	22.031±0.18	23.207±0.37	2.89	1.35	1.94	4.16	2.96	2.64

Table. S5. Stability of HPC-FITC in mouse plasma (n=5).

Tissue	Theoretical concentration (μg/mL)	24h at room temperature		Repeated freeze-thaw test for 3 times		Frozen at -20 °C for 15 days	
		Measured concentration (μg/mL)	RSD (%)	Measured concentration (μg/mL)	RSD (%)	Measured concentration (μg/mL)	RSD (%)
Heart	0.5	0.43±0.19	2.61	0.47±0.05	1.31	0.43±0.04	1.46
	5.0	4.69±0.09	3.11	4.16±0.16	4.66	4.37±0.31	2.07
	25.0	24.45±0.75	4.08	24.58±0.25	3.98	24.33±0.14	4.32
Liver	0.5	0.44±0.29	1.16	0.45±0.10	5.47	0.48±0.02	1.37
	5	4.13±0.88	5.43	4.50±0.33	1.60	4.05±0.11	3.64
	25	23.83±1.12	2.42	24.38±0.23	3.86	24.47±0.67	5.35
Splenic	0.5	0.35±0.11	4.16	0.43±0.08	6.34	0.38±0.16	2.12
	5	4.73±0.33	2.25	4.60±0.17	2.38	3.76±0.22	6.73
	25	22.32±0.23	1.38	23.98±0.84	3.21	23.96±0.80	4.98
Stomach	0.5	0.42±0.05	3.02	0.38±0.14	4.68	0.43±0.01	1.77
	5	3.91±1.24	1.31	4.69±0.40	1.89	4.66±0.26	6.26
	25	24.87±0.42	4.52	23.46±0.51	5.37	24.88±1.06	4.12
Kidney	0.5	0.38±0.13	2.63	0.36±0.07	2.42	0.39±0.05	2.86
	5	4.56±0.78	6.57	3.73±0.62	1.97	4.34±0.22	5.14
	25	23.76±0.27	4.18	24.03±1.21	5.43	23.69±0.72	1.82
Large intestine	0.5	0.47±0.03	3.54	0.42±0.13	1.88	0.46±0.11	3.12
	5	4.83±0.63	1.74	4.35±0.69	2.49	4.49±0.26	5.74
	25	24.17±0.32	5.45	24.62±0.73	5.48	24.78±0.53	1.99
Small intestine	0.5	0.43±0.29	3.82	0.39±0.11	2.16	0.41±0.09	2.08
	5	4.56±0.90	1.92	4.15±0.43	7.21	3.99±0.28	4.68
	25	23.09±0.38	6.18	24.61±0.91	4.12	23.96±0.14	3.38
Feces	0.5	0.39±0.10	2.27	0.47±0.18	2.63	0.31±0.02	1.97
	5	4.07±0.24	4.06	4.31±0.27	4.43	4.51±0.17	4.86
	25	24.85±0.31	6.77	23.82±0.35	1.62	23.74±0.51	3.51
Urine	0.5	0.45±0.04	3.32	0.37±0.04	2.14	0.40±0.13	2.51
	5	3.85±0.74	1.90	3.89±0.32	4.27	4.34±0.49	3.76
	25	23.92±0.50	5.79	23.99±0.58	1.85	24.63±0. 27	6.28
Plasma	0.5	0.43±0.13	1.03	0.41±0.09	2.36	0.44±0.08	2.49
	5	4.69±0.49	3.38	4.30±0.43	1.68	4.75±0.14	6.43
	25	24.48±0.16	2.25	24.60±1.03	4.27	23.66±0.48	5.07

Table. S6. Stability of PC1-FITC in mouse plasma (n=5).

Tissue	Theoretical concentration (μg/mL)	24h at room temperature		Repeated freeze-thaw test for 3 times		Frozen at -20 °C for 15 days	
		Measured concentration (μg/mL)	RSD (%)	Measured concentration (μg/mL)	RSD (%)	Measured concentration (μg/mL)	RSD (%)
Heart	0.5	0.38±0.05	1.24	0.35±0.11	1.48	0.33±0.03	1.49
	5.0	3.27±0.16	3.14	4.54±0.24	4.27	3.83±0.14	3.28
	25.0	23.47±0.25	6.96	23.97±0.16	3.19	23.44±0.24	5.17
Liver	0.5	0.41±0.10	1.29	0.35±0.03	1.69	0.38±0.06	1.74
	5	4.31±0.33	5.14	3.85±0.25	3.18	4.44±0.51	2.15
	25	23.94±0.23	3.16	23.84±0.64	6.19	23.71±0.32	5.16
Splenic	0.5	0.47±0.08	2.58	0.43±0.05	4.16	0.44±0.10	3.79
	5	4.28±0.17	4.57	4.00±0.47	2.25	3.94±0.16	5.17
	25	23.98±0.84	6.42	24.21±0.18	5.71	24.04±0.14	2.83
Stomach	0.5	0.38±0.14	2.14	0.32±0.07	1.57	0.40±0.02	5.73
	5	4.16±0.40	5.19	3.97±0.21	3.17	4.22±0.37	3.49
	25	24.36±0.51	6.15	23.82±0.19	5.67	24.16±0.41	7.28
Kidney	0.5	0.43±0.07	2.85	0.39±0.08	1.14	0.40±0.11	2.18
	5	4.73±0.62	4.36	4.56±0.21	3.27	4.61±0.19	4.44
	25	24.43±1.21	6.67	23.36±0.78	6.33	23.98±0.31	5.65
Large intestine	0.5	0.45±0.13	1.38	0.34±0.10	1.93	0.38±0.15	1.12
	5	3.98±0.69	2.59	3.47±0.30	5.45	4.19±0.46	3.14
	25	23.87±0.73	5.92	23.37±0.38	7.21	23.41±0.18	4.48
Small intestine	0.5	0.41±0.11	2.23	0.39±0.02	4.76	0.43±0.10	1.16
	5	4.48±0.43	1.27	3.76±0.19	3.83	4.72±0.56	3.94
	25	23.54±0.91	5.56	23.75±0.70	6.37	23.96±0.49	4.24
Feces	0.5	0.37±0.18	2.43	0.40±0.10	2.32	0.43±0.08	2.91
	5	4.71±0.27	5.32	4.26±0.64	5.32	3.75±0.37	5.08
	25	23.47±0.35	6.54	24.14±1.01	7.18	23.75±0.30	6.32
Urine	0.5	0.43±0.04	1.06	0.41±0.10	2.03	0.39±0.08	2.58
	5	4.89±0.32	2.94	4.25±0.18	5.89	3.77±0.21	5.14
	25	24.16±0.58	4.21	24.37±0.82	4.48	24.11±0.43	4.07
Plasma	0.5	0.45±0.09	3.84	0.37±0.09	1.24	0.43±0.06	2.57
	5	3.61±0.43	5.35	4.13±0.13	3.59	4.32±0.20	4.61
	25	23.81±1.03	7.76	23.93±0.27	5.10	24.08±0.54	7.17

Table. S7. Stability of PC4-FITC in mouse plasma (n=5).

Tissue	Theoretical concentration (μg/mL)	24h at room temperature		Repeated freeze-thaw test for 3 times		Frozen at -20 °C for 15 days	
		Measured concentration (μg/mL)	RSD (%)	Measured concentration (μg/mL)	RSD (%)	Measured concentration (μg/mL)	RSD (%)
Heart	0.5	0.45±0.02	1.24	0.39±0.10	3.14	0.43±0.14	3.16
	5.0	4.42±0.28	3.48	3.66±0.25	1.29	3.73±0.37	5.14
	25.0	24.76±0.43	4.57	24.02±0.69	2.58	24.84±0.28	6.96
Liver	0.5	0.41±0.06	4.36	0.43±0.16	1.38	0.46±0.10	2.85
	5	4.31±0.24	2.14	4.05±±0.30	3.23	4.27±0.51	4.21
	25	23.96±0.18	6.42	23.58±0.13	5.19	23.78±0.32	6.67
Splenic	0.5	0.43±0.10	3.46	0.35±0.06	2.64	0.39±0.11	1.06
	5	4.46±0.62	1.27	4.42±0.41	4.48	4.45±0.16	2.43
	25	24.61±0.27	5.56	23.83±0.37	6.15	24.42±0.28	5.48
Stomach	0.5	0.38±0.15	2.94	0.36±0.13	3.08	0.40±0.04	3.84
	5	4.72±0.34	5.32	4.28±0.19	5.16	4.64±0.46	4.12
	25	23.98±0.46	6.54	23.39±0.11	6.23	23.53±0.15	6.85
Kidney	0.5	0.41±0.12	1.02	0.37±0.03	1.37	0.44±0.03	2.03
	5	4.47±0.38	3.49	3.85±0.46	2.23	4.31±0.17	3.46
	25	23.87±0.57	4.56	23.26±0.26	5.69	23.44±0.24	6.01
Large intestine	0.5	0.38±0.21	1.69	0.37±0.04	2.61	0.39±0.10	3.11
	5	4.18±0.46	2.58	3.91±0.18	3.67	4.06±0.27	4.61
	25	23.87±0.39	4.99	23.31±0.64	5.34	23.68±0.19	7.89
Small intestine	0.5	0.46±0.22	2.33	0.41±0.04	2.01	0.43±0.08	2.43
	5	4.39±0.13	4.51	3.69±0.25	3.12	4.17±0.15	2.33
	25	24.48±0.49	5.63	24.12±0.64	6.54	24.31±0.40	5.12
Feces	0.5	0.45±0.18	2.94	0.38±0.07	5.89	0.40±0.16	1.06
	5	4.28±0.12	4.21	4.12±0.36	5.35	4.18±0.37	3.32
	25	23.89±0.74	6.37	23.57±0.60	3.84	23.65±0.51	5.88
Urine	0.5	0.40±0.13	2.32	0.37±0.03	1.61	0.42±0.21	1.24
	5	4.34±0.49	3.18	3.80±0.27	3.84	3.97±0.46	2.03
	25	24.68±0.27	5.47	24.14±0.23	2.16	24.63±0.82	4.81
Plasma	0.5	0.47±0.06	2.03	0.41±0.18	1.84	0.45±0.07	2.07
	5	4.24±0.18	3.41	4.06±0.48	3.48	4.15±0.68	4.86
	25	24.66±0.67	5.54	24.10±0.29	5.03	24.43±0.56	6.52
